# Reversed role of CD36 deficiency in high-fat diet or methionine/choline-deficient diet-induced hepatic steatosis and steatohepatitis

**DOI:** 10.3389/fphar.2025.1522177

**Published:** 2025-03-05

**Authors:** Wenya Zhu, Jialing Ma, Tingting Zhang, Mengmeng Zhu, Yajun Duan, Xiaoxiao Yang, Yuanli Chen

**Affiliations:** ^1^ Key Laboratory of Major Metabolic Diseases and Nutritional Regulation of Anhui Department of Education, School of Food and Biological Engineering, Hefei University of Technology, Hefei, China; ^2^ Department of Health Toxicology, Key Laboratory for Environment and Health, School of Public Health, Tongji Medical College, Huazhong University of Science and Technology, Wuhan, China; ^3^ School of Pharmacy, East China Normal University, Shanghai, China; ^4^ Division of Life Sciences and Medicine, Department of Cardiology, The First Affiliated Hospital of USTC, University of Science and Technology of China, Hefei, Anhui, China

**Keywords:** CD36, MAFLD, MASH, HFD, MCD, lipid metabolism

## Abstract

**Introduction:**

Cluster of differentiation 36 (CD36) is highly expressed in the liver of patients with metabolic dysfunction-associated fatty liver disease (MAFLD) or metabolic dysfunction-associated steatohepatitis (MASH). However, the precise role of CD36 in MAFLD/MASH is controversial. In the current study, we aimed to uncover the role of CD36 in the early stage of MAFLD/MASH induced by high-fat diet (HFD) and methionine/choline-deficient (MCD) diet.

**Methods:**

CD36^−/−^ mice and littermate control mice were fed a normal food diet (NCD); HFD or MCD diet for 6 weeks.

**Results:**

We determined that CD36 deficiency attenuated HFD-induced hepatic steatosis while exacerbating MCD diet-induced steatohepatitis. Mechanistically, CD36 deficiency reduced HFD-induced expression of fatty acid synthase (FASN), sterol regulatory element binding protein 1c (SREBP1c), and acetyl-CoA carboxylase alpha (ACC1), thereby inhibiting de novo fatty acid synthesis. The expression of superoxide dismutase and genes involving fatty acid oxidation was inhibited by MCD diet. CD36 deficiency reduced expression of genes involving fatty acid oxidation, while MCD diet had no effect on these genes expression in CD36^−/−^ mice. Meanwhile, MCD diet-reduced superoxide dismutase expression was further inhibited by CD36 deficiency. Thus, MCD-induced liver ROS and inflammation were further enhanced by CD36 deficiency. By liver lipidomic analysis, we found that the levels of triglyceride (TG), diacylglycerols (DG), acylcarnitine (AcCA), ceramide (Cer) and LPC were increased, while phosphatidylcholine/phosphatidylethanolamine (PC/PE) were decreased in MCD diet-treated CD36^−/−^ mice compared with MCD diet-treated wild type mice. Indeed, the expression of serine palmitoyltransferase 2 (SPTLC2), the key rate-limiting enzyme of ceramide synthesis, was higher in CD36^−/−^ mice.

**Discussion:**

CD36 deficiency improves HFD-induced MAFLD by inhibiting fatty acid synthesis, while accelerating MCD diet-induced MASH via promoting Cer, LPC, TG and DG accumulation to accelerate liver inflammation. The complex role of CD36 in MAFLD/MASH needs more investigation to discover the precise and effective strategy when targeting CD36.

## Graphical Abstract

**Figure:**
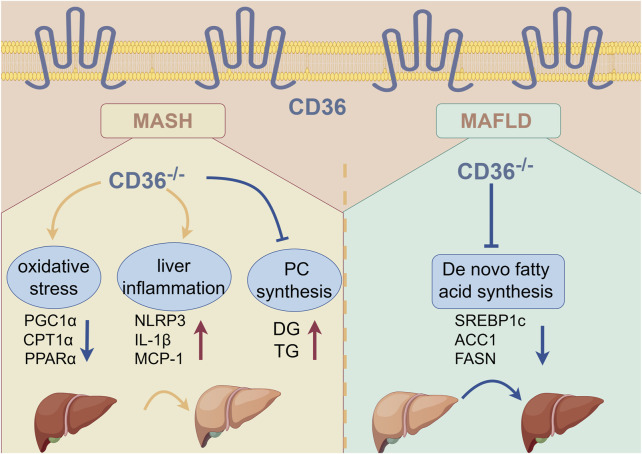
By Figdraw.

## Introduction

Metabolic dysfunction-associated fatty liver disease (MAFLD) is a major type of chronic liver disease worldwide, and its onset and progression depend on complex and unclear factors (Younossi et al., 2016). Clinically, MAFLD patients often have components of metabolic syndromes, encompassing type 2 diabetes (T2DM), obesity, hyperlipidemia, and hypertension (Younossi et al., 2019). MAFLD is a progressive liver disease that evolves from simple steatosis to its advanced stage, known as metabolic dysfunction-associated steatohepatitis or nonalcoholic steatohepatitis (MASH/NASH) (Caddeo and Romeo, 2024). The development of MASH involves excess mitochondrial oxidation and the production of free radicals, leading to the development of chronic inflammation (Lindquist et al., 2020; Gancheva et al., 2024), which initiates the liver fibrosis cascades.

Studies have shown that CD36 (a fatty acid transport protein) plays a pivotal role in MAFLD, and modulating its expression directly affects hepatic steatosis in the liver. Under physiological conditions, the expression of CD36 in hepatocytes was weak. However, under surplus lipids or the activation of specific nuclear receptor, the expression of CD36 was significantly upregulated (Steneberg et al., 2015). The expression of CD36 is regulated by various specific nuclear receptors, such as PPARs (peroxisome proliferator-activated receptors) (Maréchal et al., 2018), LXR (liver X receptor) (Lee et al., 2008), RXR (retinoid X receptor) (Han and Sidell, 2002), FXR (farnesoid X receptor) (Chen et al., 2023), and GR (glucocorticoid receptor) (Chen et al., 2022). Upon exposure to the HFD diet, primary hepatocytes and lean mouse livers exhibited enhanced uptake of free fatty acids (FFA) and increased triglycerides (TG) storage, accompanied by increased expression of CD36, ultimately accelerating the occurrence and progression of MAFLD (Koonen et al., 2007). Conversely, CD36 hepatocyte-specific knockout was protected from hepatic steatosis while improving systemic insulin sensitivity (Wilson et al., 2016). Interestingly, CD36 deficiency does not affect hepatic FFA uptake, while CD36 deficiency promotes MASH development by promoting the monocyte chemotactic protein 1 (MCP-1) expression (Zhong et al., 2017). These controversial findings suggest that the exact effect and mechanism of CD36 in the progression of MAFLD and MASH are unclear.

Imbalances in hepatic lipid metabolism may promote the progression of MAFLD/MASH (Bradbury, 2006). Lipids play a pivotal role as both structural and regulatory components, not only in energy storage molecules but also in facilitating intercellular and intracellular signaling processes, they are indispensable for the proper functioning of cellular activities (Han, 2016). Prior lipidomic investigations have revealed disruptions in the metabolism of numerous lipids, including phosphatidylcholine (PC) and phosphatidylethanolamine (PE) (Ming et al., 2017), ceramide (Cer), acylcarnitine (AcCa) and diacylglycerols (DG) (Bence and Birnbaum, 2021), during the progression of MAFLD or MASH. Studies from many years ago reported an elevation in the ratio of PE/PC within the livers of rats exposed to carbon tetrachloride (CCl_4_) (Shimizu, 1969; Sugano et al., 1970). The imbalance in the ratio of PC/PE has been implicated in triggering inflammatory responses. Notably, individuals with MASH exhibit a decreased ratio of PC/PE in their livers (Li et al., 2006). Analyzing the changes and balance of the lipidome in the liver uncovers the molecular mechanisms of MAFLD occurrence and development. In this current study, we employed a high-throughput lipidomics approach to compare the liver lipid content between CD36-knockout mice and their wild-type counterparts, both subjected to MCD diet feeding.

The role of CD36 in the initiation and progression of MAFLD/MASH was not fully understood. We used a mouse model of MAFLD/MASH by HFD or MCD diet feeding for 6 weeks. The main reason we chose these two models was to explore different disease stages and mechanisms. The 6-week HFD model is an early stage of MAFLD by providing excess fatty acids and energy, causing intrahepatic lipid accumulation. The MCD diet inhibits phospholipid metabolism through a lack of methionine and choline, thereby affecting the synthesis and secretion of very low-density lipoprotein (VLDL) (Vance and Vance, 1985; Yao and Vance, 1988), resulting in abnormal accumulation of lipids in the liver and further inducing severe inflammation and liver damage. Choosing a combination of these two models can provide a comprehensive view of CD36 function in different metabolic contexts. This experimental work aimed to elucidate the role of CD36 in the development of MAFLD/MASH under the HFD/MCD model.

## Materials and methods

### Reagents

Rabbit anti-peroxisome proliferator-activated receptor alpha (PPARα), interleukin 1β (IL-1β), sterol regulatory element binding transcription factor 1c (SREBP1c) and β-actin polyclonal antibodies were purchased from Abclonal (Boston, United States). Mouse anti-α-smooth muscle actin (SMA) monoclonal antibody, rabbit anti-peroxisome proliferators-activated receptor γ coactivator l alpha (PGC1α) and carnitine palmitoyltransferase 1 (CPT1α) polyclonal antibodies were obtained from Affinity Biosciences (Cincinnati, United States). Anti-cluster of differentiation 36 antibody were obtained from Proteintech Group (Chicago, United States). Rabbit anti-fatty acid synthesis (FASN), superoxide dismutase 1 or 2 (SOD1/SOD2), heat shock protein 90 (HSP90), and glyceraldehyde-3-phosphate dehydrogenase (GAPDH) polyclonal antibodies were acquired from Proteintech Group (Chicago, United States). Rabbit anti-acetyl-CoA carboxylase alpha (ACC1) polyclonal antibody was purchased from Cell Signaling Technology (Danvers, MA, United States). LabAssay Triglyceride assay kit was purchased from WAKO (Kanagawa Prefecture, Japan). HFD (35% fat and 2.5% cholesterol) and MCD diet were purchased from Medicience (Jiangsu, China). Dihydroethidium (DHE) was acquired from MedChemexpress (New Jersey, United States). Unless specified otherwise, all remaining reagents were procured from Sigma-Aldrich.

### *In vivo* experiment

The Ethics Committee of Hefei University of Technology has granted approval for all animal study protocols and conformed to the Guide for the Care and Use of Laboratory Animals published by NIH. The animal studies adhered to the ARRIVE guidelines (Percie du Sert et al., 2020). CD36^−/−^ mice with C57BL/6J background were generated by the GemPharmatech Co., Ltd. using the CRISPR/Cas9 technology to knockout the exon4 of *CD36* transcript. The offspring were genotyped by PCR, with the following primer sequences: Cd36-5wt-F: 5′-TTT​CCC​TAA​GAC​TCT​GCT​ACT​ATT​T-3′; CD36-5wt-R: 5′-ATG​CAA​AAT​CAT​TTT​AGC​TCT​GTG-3′; CD36-wt-F: 5′-TCC​AGC​AAT​CCT​CAA​ACA​TA-3′; Cd36-wt-R: 5′-CCTTTGGCAACACTCCCTTA-3′. CD36^−/−^ mice and littermate control mice (male, ∼8 weeks old) were randomized into three groups (n = 6): control group, mice were fed normal chow diet (NCD); HFD or MCD group, mice were fed HFD or MCD diet respectively. After 6 weeks, all mice were anesthetized and euthanized in a CO_2_ chamber, following by the collection of blood and liver samples for further analysis. Mice serum was prepared and used to measure the lipids profile and liver function parameters employing commercial kits sourced from Medicalsystem Biotechnology Co., Ltd. (Zhejiang, China). The analysis was conducted using the HITACHI Automatic Aralyzer (3100).

### Western blot analysis

Total proteins were extracted from a segment of liver, then the expression of CD36, SREBP1c, FASN, ACC1, PGC1α, CPT1α, PPARα, SOD1, SOD2, SMA, and IL-1β were detected by Western blot (Chen et al., 2015). Briefly, protein was extracted from liver samples using RIPA lysis buffer. After determination of protein concentration by BCA (bicinchoninic acid) methods, equal protein in each sample was then separated by SDS-PAGE and transferred onto NC membranes. The membranes were blocked with 5% skim milk (prepared in PBST) at room temperature for 1 h to prevent nonspecific binding. Subsequently, the membranes were cut into appropriate sizes based on the molecular weight of the target proteins. The membranes were incubated with specific primary antibodies at 4°C overnight. After washing with PBST 3 times (5–10 min each) to remove unbound antibodies, the membranes were incubated with HRP-conjugated secondary antibodies at room temperature for 1 h. The membranes were washed again with PBST 3 times (5–10 min each). Images were captured using a chemiluminescence imaging system (Qinxiang, ChemiScope 3300 Mini, China). The band intensities were quantified using ImageJ software. The density of the target protein bands in each sample was normalized to HSP90, GAPDH, or β-ACTIN to reduce variance. The quantitative analysis were conducted across with three biological repeated experiments, and the representative images were shown.

### Quantitative real time-PCR (qPCR)

Total RNA was extracted from approximately 30 mg of liver tissue using TRIzol reagent (Invitrogen, Carlsbad, CA, United States of America). Then, 1 μg total RNA was used to reverse-transcribe into cDNA, followed by quantitative PCR with a SYBR Green PCR master mix (Vazyme Biotech, Nanjing, China) along with the specific primers listed in Table 1. The expression levels of PGC1α, PPARα, NLRP3, IL-1β, MCP1, SPTLC1 and SPTLC2 mRNA were normalized to GAPDH mRNA in the corresponding samples (He et al., 2023).

**TABLE 1:** The sequences of primers for qPCR analysis.

Gene	Forward	Reverse
m-PPARα	AGT​TCG​GGA​ACA​AGA​CGT​TG	CAG​TGG​GGA​GAG​AGG​ACA​GA
m-GAPDH	GGT​GGT​CTC​CTC​TGA​CTT​CAA​CA	GTT​GCT​GTA​GCC​AAA​TTC​GTT
m-PGC1α	CCC​TGC​CAT​TGT​TAA​GAC​C	TGC​TGC​TGT​TCC​TGT​TTT​C
m-NLRP3	ATT​ACC​CGC​CCG​AGA​AAG​G	TCG​CAG​CAA​AGA​TCC​ACA​CAG
m-MCP-1	CAG​CCA​GAT​GCA​GTT​AAC​GC	GCC​TAC​TCA​TTG​GGA​TCA​TCT​TG
m-IL-1β	GAC​CTT​CCA​GGA​TGA​GGA​CA	AGC​TCA​TAT​GGG​TCC​GAC​AG
m- SPTLC1	AGT GGTGGGAGAGTCCCTTT	CAG​TGA​CCA​CAA​CCC​TGA​TG
m- SPTLC2	GGA​TAC​ATC​GGA​GGC​AAG​AA	ACC​TGG​TGT​TCT​CAG​CCA​AC

### Oil Red O, hematoxylin and eosin (H&E), and sirius red staining

Part of the liver tissue was collected and fixed in 4% paraformaldehyde for 24 h, followed by preparing frozen and paraffin sections (Chen et al., 2015). To detect lipid content, the frozen section was subjected to 0.5% Oil Red O solution for 30 min. After washed twice with 60% isopropanol, the sections were stained with hematoxylin for the nucleus. Then, the sections were sealed with glycerin and photographed with a microscope (Zeiss, Germany). Meanwhile, a piece of fresh liver tissue was used to determine TG level by LabAssay Triglyceride assay kit (Chen et al., 2015).

The paraffin sections were conducted H&E and Sirius Red Staining as previously described (Li et al., 2021), which were used to study morphology and collagen content in the liver, respectively.

### Determination of ROS levels

The level of superoxide in the liver was determined using dihydroethidium (DHE) staining, as described previously (Li et al., 2024). Briefly, frozen sections were incubated with a solution of PBS containing DHE (final concentration of 5 μM) for 30 min. After the sections were washed 3 times with PBS, all sections were mounted with an anti-fade mounting medium. The images were captured by Zeiss fluorescence microscope (Qiao et al., 2019).

### Lipidomic analysis by LC-MS/MS

Liver lipids were extracted according to MTBE (Methyl tert-butyl ether) method as described previously (Lyn-Cook et al., 2009). The lipidome was determined as described previously (Zhou et al., 2023). “Lipid Search” was used to identify the lipid species based on MS/MS math. Both mass tolerance for precursor and fragment were set to 5 ppm.

### Statistical analysis

All experiments were repeated at least three times (biological duplication), and the values are presented as means ± SEM. GraphPad Prism 7.0 software was used to perform statistical analysis on the data. The statistical analysis was performed with one-way analysis of variance (ANOVA) followed by *post hoc* Bartlett’s test (more than two groups) or unpaired Student’s t-test (two groups). For all tests, the significant differences were considered if p < 0.05.

## Results

### CD36 deficiency aggravates MCD-induced MASH while improving HFD-induced MAFLD

**FIGURE 1:**
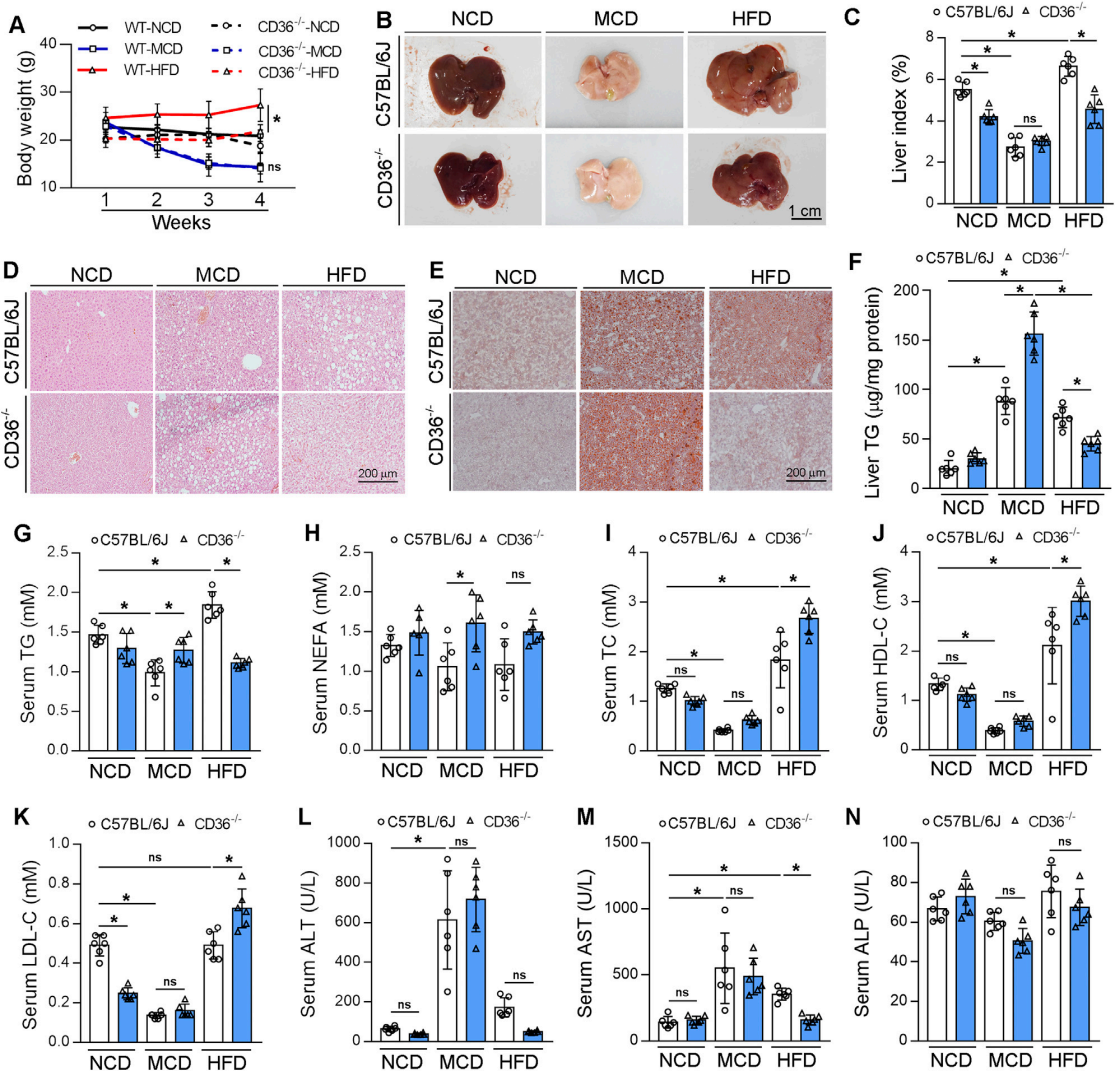
CD36 deficiency aggravates MCD diet-induced MASH, but improves HFD-induced MAFLD. C57BL/6J wild-type (WT) or CD36^−/−^ mice (male, ∼8-week-old) were randomly divided into 3 groups (6/group) which were fed with normal chow diet (NCD), HFD or MCD diet for 6 weeks, and used for the following assays: **(A)** body-weight (weekly); **(B)** liver photos; **(C)** liver index (the ratio of liver weight to body weight); **(D)** Hematoxylin and eosin (H&E) staining; **(E)** Oil Red O staining; **(F)** TG quantitative analysis with total liver lipid extract. **(G–N)** Serum TG, NEFA, TC, HDL-C, LDL-C, ALT, AST and ALP levels. *P < 0.05; ns, no significance (n = 6).

The function of CD36 in liver lipid metabolism is controversial. It is reported that CD36 enhanced fatty acid uptake and biosynthesis in hepatocytes (Zeng et al., 2022). However, when CD36 is specifically knocked out in the liver of mice, these animals exhibit resistance to the development of liver steatosis induced by an HFD (Zeng et al., 2022). However, it is also reported that CD36 deficiency enhanced HFD-induced liver steatosis by activating the transcription of MCP-1 in hepatocytes (Zhong et al., 2017). To explore the role of CD36 in MAFLD/MASH, we fed CD36^−/−^ mice and littermate control mice with NCD, HFD or MCD diet for 6 weeks. CD36 deficiency reduced HFD-increased weight gain induced by HFD but did not affect the weight loss caused by MCD diet (Figure 1A). HFD enlarged liver size and weight which was attenuated by CD36 deficiency (Figures 1B, C). CD36 deficiency also reduced liver weight under NCD (Figures 1B, C). Surprisingly, the livers of CD36^−/−^ mice fed with an MCD diet became whiter, implying the increased accumulation of lipids (Figure 1B). H&E staining shows that CD36 deficiency enhanced MCD-induced vacuolar lesions while reduced HFD-induced liver steatosis (Figure 1D). Oil red O staining and triglyceride (TG) quantitative analysis also confirmed the conclusion that CD36 deficiency had reversed function on HFD or MCD diet-induced liver steatosis (Figures 1E, F). CD36 knockout reduced serum TG level but not non-esterified fatty acids (NEFA) level under HFD. However, serum TG and NEFA levels were increased by CD36 knockout under MCD diet feeding (Figures 1G, H). Serum total cholesterol (TC), low-density lipoprotein cholesterol (LDL-C) and high-density lipoprotein cholesterol (HDL-C) were increased in CD36^−/−^ mice fed with HFD, while did not affect lipids profile in CD36^−/−^ mice fed with MCD (Figures 1I–K). Serum LDL-C level was reduced in CD36^−/−^ mice fed with NCD (Figure 1K). Simultaneously the elevated levels of aspartate aminotransferase (AST) and alanine aminotransferase (ALT), the key markers of hepatotoxicity, were detected in MCD-fed CD36^−/−^ mice, suggesting that liver damage occurred (Figures 1L, M). The alkaline phosphatase (ALP) level was not changed among the groups (Figure 1N). In summary, CD36 deficiency inhibited HFD-induced MAFLD but accelerated MCD-induced MASH.

### CD36-deficiency alleviated HFD-induced MAFLD by reducing hepatic fatty acid synthesis, but aggravated MCD-induced MASH by increasing oxidative stress

**FIGURE 2:**
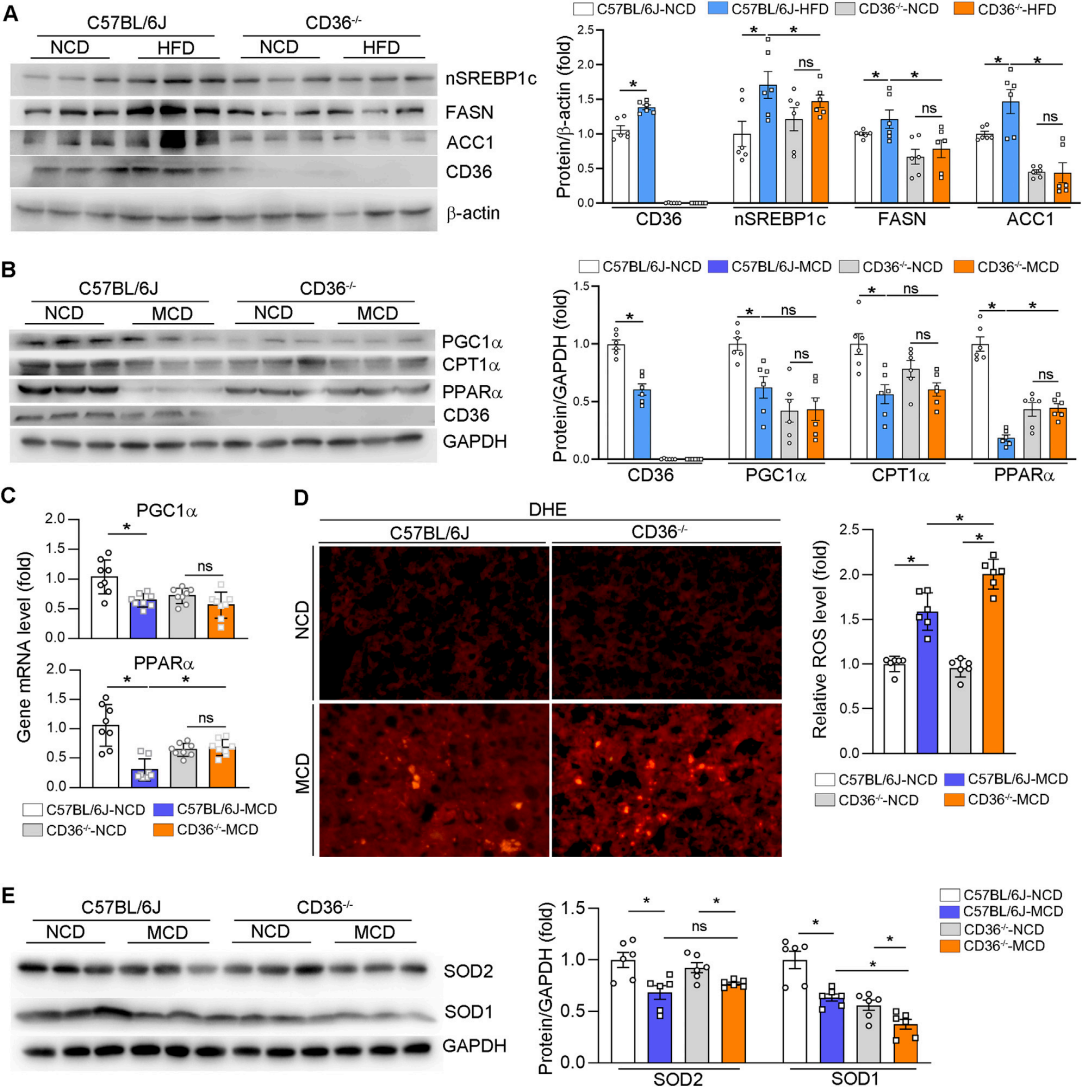
CD36-deficiency reduces HFD-induced hepatic fatty acid synthesis, but aggravates MCD-induced oxidative stress. **(A–C, E)** Total RNA and proteins were extracted from a piece of liver. The proteins expression of nSREBP2, FASN, ACC1, CD36 **(A)**, PGC1α, CPT1α, PPARα, CD36 **(Β)**, SOD1 and SOD2 **(E)** were determined by Western blot. The mRNA expression of PGC1α and PPARα were determined qPCR **(C)**. **(D)** ROS in the liver was determined by Dihydrothidium (DHE) staining. *P < 0.05; ns, no significance (n = 6).

A previous study showed that lipogenic genes expression were significantly suppressed in hepatic CD36-specific knockout mice, while the HFD model exacerbates MAFLD mainly by up- or downregulation of the expression of genes encoding proteins or enzymes involved in adipogenesis (Yang et al., 2010). Therefore, we mainly examined the expression of CD36, SREBP1c, ACC1 and FASN in HFD-fed mice. CD36 expression was significantly increased in HFD-fed wild-type mice (Figure 2A). The protein levels of lipogenic genes (nuclear SREBP1c, FASN and ACC1) were significantly increased by HFD in wild-type mice, which were substantially reduced in the CD36^−/−^ mice liver (Figure 2A), suggesting that CD36 deficiency could attenuate hepatic lipid deposition by inhibiting lipogenesis in HFD-induced MAFLD.

Hepatic β-oxidation is involved in the MCD-induced MASH model (Liao et al., 2024). CD36 expression was decreased in MCD-fed wild-type mice (Figure 2B). We detected that the expression of proteins and RNAs involved in β-oxidation (PGC1α, CPT1α, and PPARα) in the liver were downregulated by MCD diet in wild-type mice (Figures 2B, C). CD36 deficiency can reduce the basal level of these genes, but the inhibitory effect of MCD diet on these genes was abolished in CD36^−/−^ mice (Figures 2B, C), suggesting that CD36 deficiency increased lipid accumulation in MCD-induced MAFLD (Figures 2B, C). Since lipid accumulation promotes mitochondrial dysfunction that eventually leads to oxidative stress. We examined ROS levels in the liver, as shown by DHE staining in Figure 2D, MCD-fed CD36^−/−^ mice had significantly enhanced ROS levels in the liver than MCD-fed C57BL/6J mice. It is well known that SOD1 and SOD2 are antioxidant enzymes that protect cells from ROS-induced damage (Farrell et al., 2019). Therefore, we detected the expressions of SOD1 and SOD2 and found that the protein expression of SOD was decreased in MCD-fed CD36^−/−^ mice (Figure 2E), which suggested that the increased ROS level might be due to the decreased SOD activity. Collectively, it was demonstrated that CD36 deficiency can exacerbate MASH under MCD diet by increasing oxidative stress in the liver.

### CD36 deficiency may exacerbate MAFLD/MASH under MCD diet by increasing liver inflammation

**FIGURE 3:**
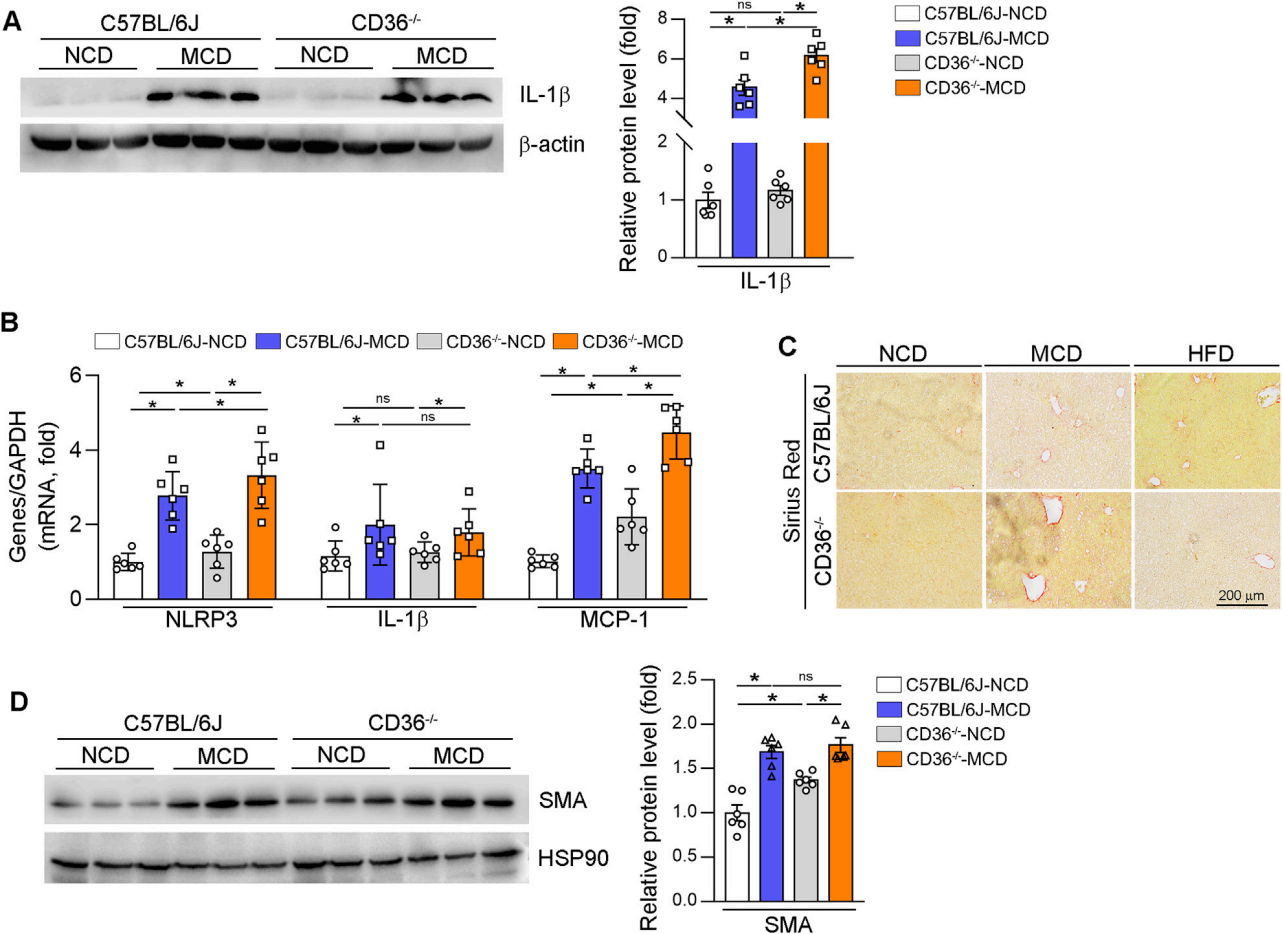
CD36 deficiency exacerbates MCD-induced MASH by increasing liver inflammation. **(A, B, D)** Total RNA and proteins were extracted from a piece of liver. The protein expression of IL-1β **(A)** and SMA **(D)** was determined by Western blot. The mRNA expression of NLRP3, IL-1β and MCP1 was determined by qPCR **(B)**. *P < 0.05; ns, no significance (n = 6). **(C)** Liver fibrosis was determined by Sirius Red staining with liver paraffin sections.

Oxidative stress is identified as a trigger to activate the NLRP3 inflammasome, which in turn promotes increased expression of more pro-inflammatory factors (Farrell et al., 2019). To determine whether CD36 contributes to the expression of the NLRP3 inflammasome, we conducted an analysis of the expressions of NLRP3, IL-1β, and MCP-1 in the livers of mouse and found that CD36 deficiency activates IL-1β and NLRP3 expression (Figures 3A, B). The expression of MCP-1 was further upregulated in MCD-fed CD36^−/−^ mice liver (Figure 3B). In addition, we conducted to assess the impact of CD36 on liver fibrosis. Although 6-week MCD diet feeding caused moderate liver fibrosis, sirius red staining data indicate that liver fibrosis was more severe in MCD-fed CD36^−/−^ mice compared to MCD-fed wild-type mice (Figure 3C). CD36 deficiency increased SMA protein in NCD, which was upregulated in CD36^−/−^ mice (Figure 3D). The results above demonstrate that CD36 may exacerbate MAFLD/MASH under the MCD diet by increasing liver inflammation.

### CD36 deficiency affects PC synthesis and eventually accelerates MCD-induced MAFLD/MASH in MCD-fed mice

**FIGURE 4:**
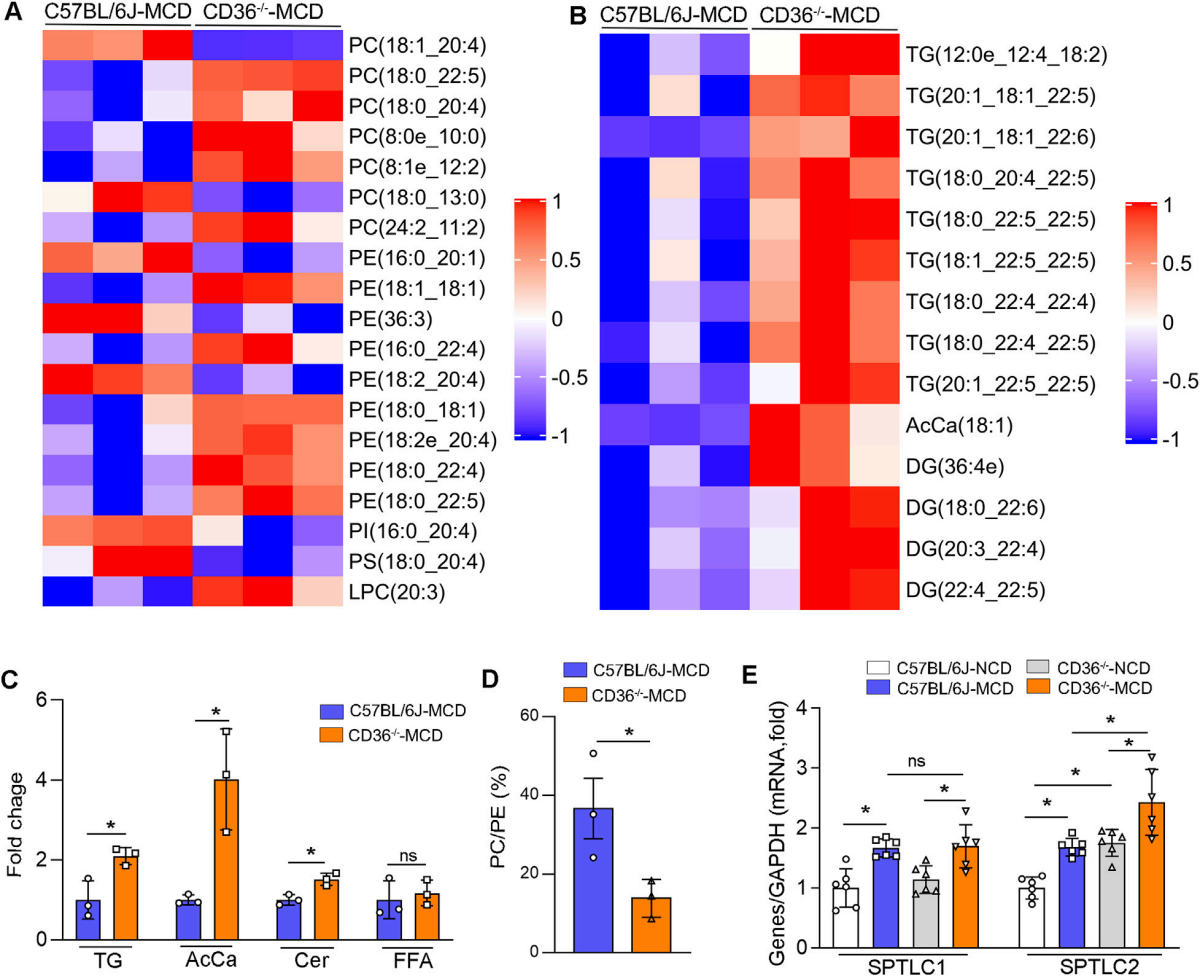
CD36 deficiency affects GP remodeling and Cer synthesis to aggravate MCD-induced MASH **(A–D)** Metabolomic analysis of liver samples from MCD-treated C57BL/6J and CD36^−/−^ mice (n = 3). Relative abundance of PC, PE, PI, PS, LPC **(A)**, TG, DG and AcCa **(B)** in the liver. **(C)** Alterations of TG, AcCa, Cer and FFA levels. **(D)** The ratio of total PC to PE in the liver. *P < 0.05; ns, no significance (n = 3). **(E)** The mRNA expression of SPTLC1 and SPTLC2 in liver tissues was determined by qPCR. *P < 0.05; ns, no significance (n = 6).

Given the pivotal roles that lipid metabolism disorders play in numerous biological processes, including cell proliferation, apoptosis, and inflammation (Blumberg et al., 1995), we performed LC-MS-based lipidomic analysis of the lipid profile of liver samples and detected 43 lipids classes, which include PC, PE, TG, DG, AcCA, FA, and Cer, etc (Figures 4A, B). We found that the levels of TG, AcCA, and Cer were increased in MCD-fed CD36^−/−^ mice (Figure 4C). At the same time, the PC/PE ratio in the liver homogenate was reduced (Figure 4D), which means that the membrane integrity was broken and eventually led to liver damage (Garcia-Ruiz et al., 2015). The changes in lipidomics indicate that the development and occurrence of MAFLD/MASH were aggravated in MCD-fed CD36^−/−^ mice. Then we found that the content of ether-linked PC (8:0e-10:0), PC (8:1e-12:2), PE (18:2e-20:4) and the content of PUFA-PE increased (Figure 4A), which further validates our experimental results. SPTLC1 and SPTLC2, the key rate-limiting enzyme of Cer synthesis, were increased in MCD-fed mice, and SPTLC2 was higher in MCD-fed CD36^−/−^ mice than that in C57BL/6J mice (Figure 4E). This suggests that the accumulation of Cer could cause oxidative stress and ultimately lead to liver damage. At the same time, the content of DG and TG also increased (Figure 4B). The results in Figure 4 suggest that CD36 deficiency may affect GP remodeling and Cer synthesis and ultimately lead to TG accumulation and aggravate the occurrence of MASH/MAFLD.

## Discussion

The role of CD36 in MAFLD/MASH is controversial. The exact effect and mechanism of CD36 in the initial stage of MAFLD and MASH are unclear. In the present study, we chose two models to explore the mechanisms of CD36 on different disease stages of MAFLD and MASH. We found that hepatic steatosis was alleviated in CD36^−/−^ mice fed with HFD. In contrast, MCD-fed CD36^−/−^ mice developed severe TG accumulation, liver damage, and liver inflammation. Previous research has shown that clinically CD36-deficient patients exhibit hyperlipidemia, insulin resistance, fatty liver, and atherosclerosis (Hirano et al., 2003). CD36 deletion exacerbates steatosis by impairing hepatic triglyceride secretion in ob/ob mice (Janabi et al., 2000). CD36-deficient mice have decreased hepatic insulin sensitivity when given an HFD diet (Goudriaan et al., 2003). Hepatocytes or Kupffer cells specific deletion of CD36 alleviates steatosis in HFD-fed mice (Fang et al., 2020). The inconsistent data demonstrate that CD36 is important in liver lipid metabolism both in physiological and pathological conditions. The exact role of CD36 in liver steatosis may depend on other complications such as obesity, diabetes, chronic inflammation, insulin resistance and maybe cardiovascular disease. In the HFD model, we mainly explored the role of CD36 loss on proteins related to fatty acid synthesis, which is the main process in HFD-induced MAFLD. We disclosed that CD36 deficiency reduced genes involved in lipogenesis in HFD-induced MAFLD. However, we did not explore the β-oxidation and inflammation levels in the HFD model, which should be considered in the future.

In the MCD diet-induced MASH model, methionine deficiency causes liver damage, inflammation, mitochondrial damage and fibrosis, while choline deficiency causes bullous steatosis (Lin et al., 2020). Studies have shown that mitochondrial β-oxidation constitutes the primary catabolic route for the majority of NEFA within the liver (Lin et al., 2020), and the nuclear transcriptional factor PPARα orchestrates the regulation of key genes essential for diverse fatty acid oxidation reactions, while CPT1α is an important rate-limiting enzyme in the β-oxidation process (Pawlak et al., 2015). Our research has uncovered a significant downregulation in the expressions of PPARα, CPT1α and PGC1α in CD36^−/−^ mice, which suggested that the fatty acid oxidation capacity in CD36-deficient mice, within the context of the MCD model, is notably compromised. Commonly employed concentrations of CPT1α inhibitors have been shown to elicit severe oxidative stress (Jiang et al., 2022), which, along with the associated inflammation, are pivotal factors that expedite the progression from steatosis to more advanced conditions such as MASH or MAFLD (Du et al., 2019). Excessive ROS accumulation triggers the activation of the inflammasome NLRP3, which upregulates the production of the pro-inflammatory cytokine IL-1β. MCP-1 is also critical for the development of liver inflammation, the production of oxidative stress, and the progression towards fibrosis in the MCD diet-induced MASH model (Baeck et al., 2012; Li et al., 2015). Here, we found that the accumulation of ROS in the liver of MCD diet-fed CD36^−/−^ mice was more serious than that of MCD diet-fed C57BL/6J mice, and the expression of SOD1 was significantly inhibited, implying severe oxidative stress occurred in MCD diet-fed CD36^−/−^ mice. Accordingly, CD36 deficiency activated the expression of pro-inflammatory factors NLRP3 and MCP-1, while the expression of inflammatory factors was increased under the MCD diet. Moreover, liver fibrosis was exacerbated in the liver of MCD diet-fed CD36^−/−^ mice. Our data suggest that CD36^−/−^ mice aggravated liver injury by stimulating oxidative stress and associated inflammatory cytokine release on the MCD diet.

Recently, lipidomics has been widely used to study lipid dysfunction. The hepatic abnormal lipid metabolism could lead to a variety of diseases including MASH/MAFLD (Shi et al., 2016). GPs are the major lipids in cell membranes, whereas PC and PE are the two most abundant phospholipids (Ma et al., 2021). PC biosynthesis occurs through two pathways, 70% of which are derived from the CDP-choline pathway (Ling et al., 2012), and 30% derived from the methylation synthesis of PE (Ling et al., 2012). Hepatic PC/PE level may be a common feature of liver inflammation. A decreased PC/PE ratio impairs endoplasmic reticulum calcium homeostasis and further contributes to ER stress in ob/ob mice (Fu et al., 2011). We found that the ratio of PC/PE decreased in MCD diet-fed CD36^−/−^ mice, while the content of AcCa, Cer and total TG increased in the liver of these mice. Additionally, the increase in PC containing ether linkages and PUFAs and the decrease in PCs from SFAs indicate that the saturation of fatty acyl chains has changed, and the PCs containing PUFAs and ether linkages lead to cells more prone to peroxide reactions, which eventually lead to oxidation stress. This also explains the severe accumulation of ROS in MCD diet-fed CD36^−/−^ mice. At the same time, LPC is an effective inflammatory lipid, and its high expression is related to liver injury (Luo et al., 2018). In our study, the expression of LPC (20:3) was increased, which also explained that MCD diet-fed CD36^−/−^ mice cause increased inflammation.

Previous research has shown that abnormal Cer level leads to induction of signaling pathways that promoting hepatic steatosis *in vitro* and *in vivo* (Holland and Summers, 2008). Ceramides are kind of lipid signaling factors which involved in the process of oxidative stress. In animal models, hepatic free fatty acids are increased due to inflammation and fat intake, when storage capacity is exceeded, free fatty acids can form ceramides, ultimately leading to the deposition of TG in the liver (Gadgil et al., 2022). SPTLC is a key enzyme involved in ceramide biosynthesis. Inhibition of SPTLC activity in mice could reduce ceramide levels, thereby improving lipid profiles and preventing the onset of atherosclerosis and diabetes (Glaros et al., 2008). High ceramide concentrations being associated with liver inflammation as a marker for the diagnosis of MASH (Summers, 2020). Our study revealed an increase in hepatic Cer content and a significant upregulation of SPTLC2 expression in MCD diet-fed CD36 knockout (CD36^−/−^) mice compared to MCD diet-fed C57BL/6J mice. This suggests that CD36 could affect liver injury by increasing Cer biosynthesis under MCD diet.

Collectively, our study demonstrates the dual effect of CD36 on MAFLD/MASH under HFD and MCD diets in which CD36 deficiency alleviated HFD-induced MAFLD by attenuating lipogenesis, but had the opposite effect on MCD-induced MASH. CD36 deficiency can cause lipid metabolism disturbance by activating inflammation, oxidative stress, and ultimately liver damage in MCD diet. The complex role of CD36 in MAFLD/MASH needs more investigation to discover the precise and effective strategy when targeting CD36.

## Data Availability

The original contributions presented in the study are included in the article/Supplementary Material, further inquiries can be directed to the corresponding author.

## Abbreviations

ACC1, acetyl-CoA carboxylase alpha; AcCa, acylcarnitine; Cer, ceramide; DG, diacylglycerols; FASN, fatty acid synthase; FFA, free fatty acids; GP, glycerophospholipids; HFD, high-fat diet; IL-1β, interleukin 1beta; MAFLD, metabolic dysfunction-associated fatty liver disease; MASH, Metabolic dysfunction-associated steatohepatitis; MCP-1, monocyte chemotactic protein 1; MCD, methionine/choline-deficient; PC, Phosphatidylcholine; PE, phosphatidylethanolamine; SREBP1c, sterol regulatory element binding protein 1c; SPTLC2, serine palmitoyltransferase 2; TG, Triglyceride.
