# Supplementary figures and images for: Reversed role of CD36 deficiency in high-fat diet or methionine/choline-deficient diet-induced hepatic steatosis and steatohepatitis
Source: Front Pharmacol. 2025 Mar 5;16:1522177. doi: 10.3389/fphar.2025.1522177 (PMC11919839; doi:10.3389/fphar.2025.1522177)

Fig.2A

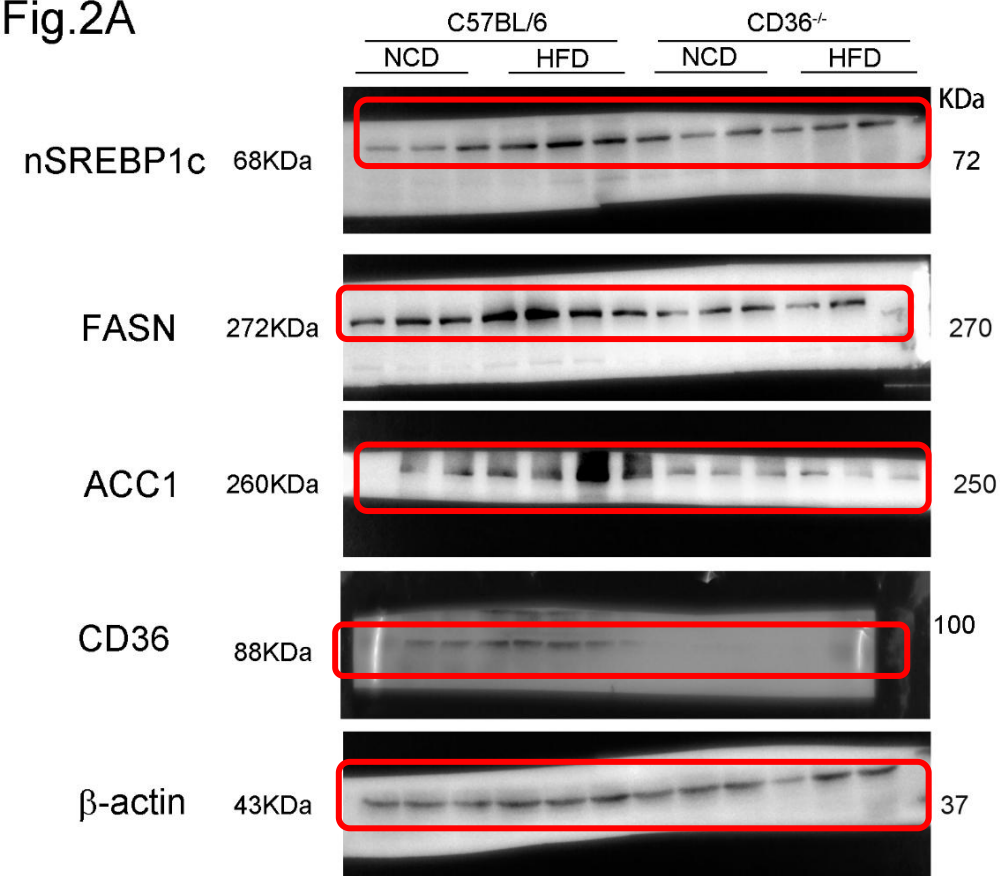

Fig.2B

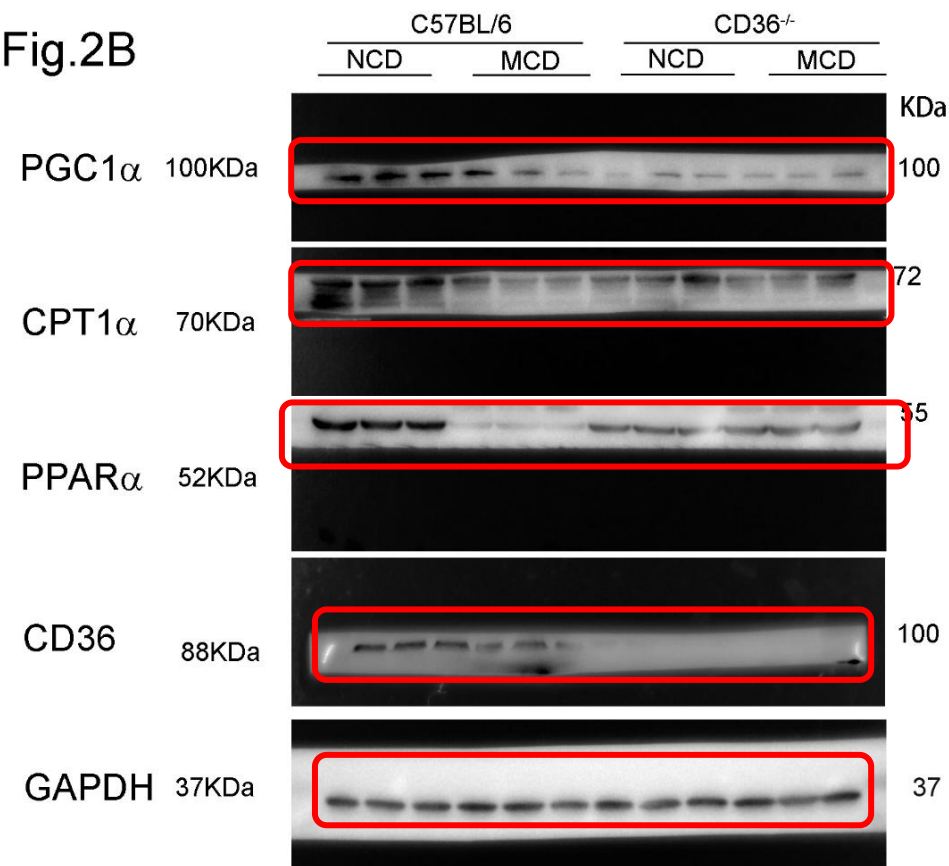

Fig.2E

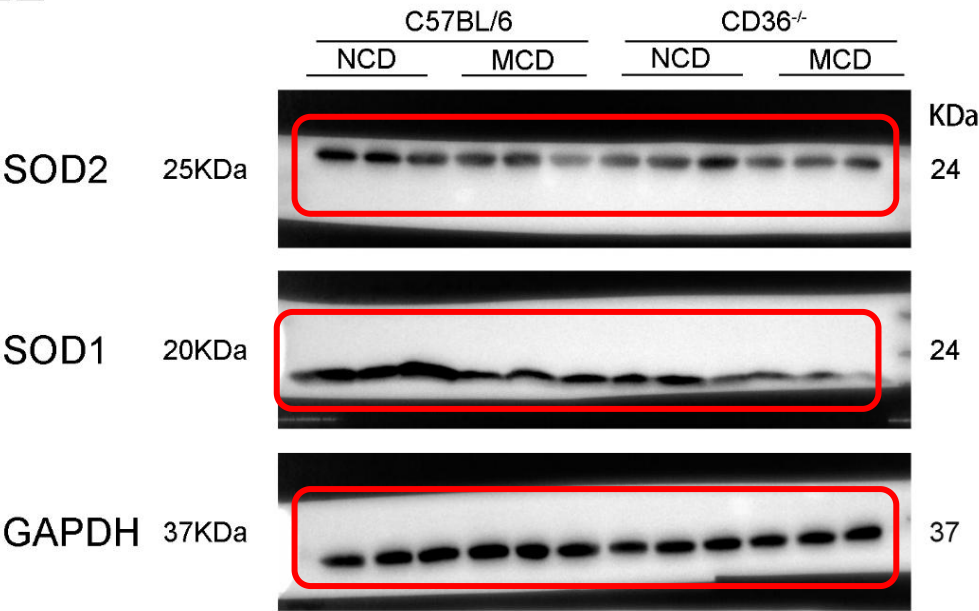

Fig.3A

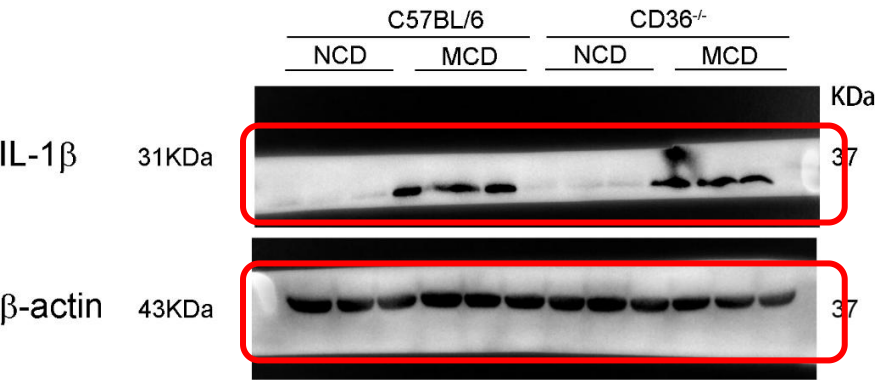

Fig.3D

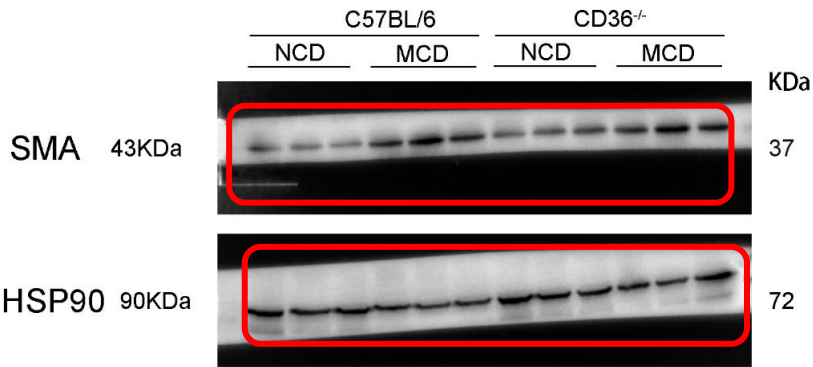

Supplement: Supplementary file 1 [file DataSheet1.PDF]
